# Electromagnetically induced polarization grating

**DOI:** 10.1038/s41598-018-21494-8

**Published:** 2018-02-15

**Authors:** Lu Zhao

**Affiliations:** School of Physics, Beihang University, Beijing, 100191 China

## Abstract

Based on electromagnetically induced transparency (EIT), we investigate an all-optical grating structure to realize polarization-dependent multiple beam splitting in the Raman-Nath limit. To optimize the grating performance, higher excited state [e.g., *nS*_1/2_ (*n* ≥ 6)] of ultracold ^87^Rb atoms is employed to construct a five-level Ξ-Λ system sharing one common populated ground state. A principal advantage of our proposed scheme is that the *σ*^±^ components of a linearly polarized weak probe field can be decoupled and thus be independently diffracted with high efficiency in both one and two dimensions by exploiting different quasi-standing waves as the two strong coupling fields in the Ξ and Λ configurations. Such an all-optical polarization-sensitive operation could greatly enhance the tunability and capacity of all-optical multiplexing, interconnecting, and networking in free space for both classical and quantum applications.

## Introduction

The effect of electromagnetically induced transparency (EIT) offers opportunities to control light with light, whereby all-optical devices can be implemented to significantly improve the performance of optical information processing systems in both classical and quantum regimes^1–3^. As an important application of EIT effect, electromagnetically induced grating (EIG) has been actively studied for all-optical multichannel devices^4–11^. Due to the diffraction effect, a weak probe laser can be transversely split into a series of high-order components by strong standing-wave (SW) coupling fields in both one and two dimensions. To further increase the diffraction efficiency in the far field, various periodic structures, such as image-induced blazed gratings^12–14^, sinusoidal phase gratings^15^, and volume holographic gratings^16,17^, have been developed in atomic ensembles, which can extend the functionality of EIG for realistic applications. Also, the diffraction gives rise to other interesting optical phenomena, such as near-field Talbot self-imaging for ultracold atoms^18,19^.

As a fundamental property, the polarization degree of freedom can strongly influence the propagation dynamics of light in EIT media, which plays a vital role in quantum information processing. For example, all-optical polarization phase gates could be created in an atomic system to realize a conditional phase shift of the order of *π* for quantum logic devices^20–22^. Moreover, controllable storage and retrieval of polarization qubit in EIT-related atomic systems could be a promising candidate to implement optical quantum memory, which proves to be a crucial operation for a global quantum network^23–30^. Therefore, a further study of polarization-dependent EIG structures for multiple beam diffraction may help advance the development of all-optical multichannel processing for photon polarization information in free space.

In this article, we present an electromagnetically induced polarization grating (EIPG) scheme to realize polarization-resolved multiple beam diffraction for a weak probe laser in the Raman-Nath limit. By involving multiple Zeeman sublevels in 5*S*_1/2_, 5*P*_1/2_, and *nS*_1/2_ (for example *n* = 10) states of an ultracold ^87^Rb atomic ensemble, a five-level Ξ(cascade)-Λ system is examined under the condition of EIT. Two sets of thin grating structures can be simultaneously but independently induced by using different quasi-SWs as the coupling fields for the Ξ and Λ configurations, respectively. By dynamically tuning the optical parameters of the EIT system, the two circularly polarized *σ*^±^ components of the probe field can be decoupled and flexibly controlled by diffraction, thus generating a plurality of purely polarized beams at different angles in one and two dimensions in the far field.

## Methods

### Light-atom interactions in EIPG

**Figure 1:**
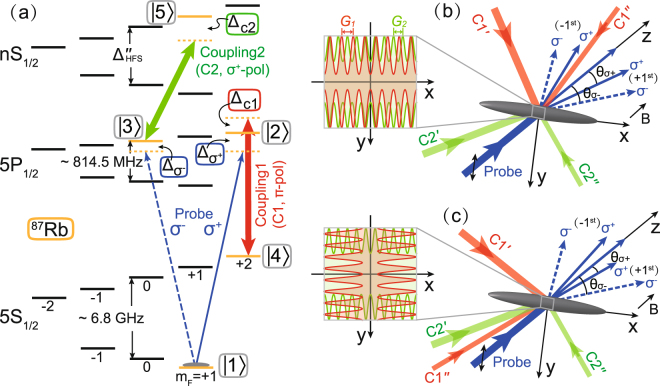
(**a**) Schematic of the five-level Ξ-Λ system in ultracold ^87^Rb atoms. The five states correspond to |5*S*_1/2_, *F* = 1, *m*_*F*_ = +1〉 (|1〉), |5*P*_1/2_, *F* = 2, *m*_*F*_ = +2〉 (|2〉), |5*P*_1/2_, *F* = 2, *m*_*F*_ = 0〉 (|3〉), |5*S*_1/2_, *F* = 2, *m*_*F*_ = +2〉 (|4〉), and |*nS*_1/2_, *F* = 2, *m*_*F*_ = +1〉 (|5〉), where *n* ≥ 6. The single-photon detunings ${{\rm{\Delta }}}_{{\sigma }^{+}}$, ${{\rm{\Delta }}}_{{\sigma }^{-}}$, Δ_C1_, Δ_C2_ are defined as the corresponding laser frequencies minus the corresponding transition frequencies. Also shown are the possible experimental setups for one-dimensional (**b**) and two-dimensional (**c**) EIPGs. In (**b**), two laser beams (C1′ and C1″) interfere to form the quasi-SW C1 field oriented in the *x* direction, and two laser beams (C2′ and C2″) interfere to form the quasi-SW C2 field oriented in the *x* direction as well. The spatial period of the C1 (C2) field is *G*_1_ (*G*_2_). The diffracted *σ*^±^ beams are all in the *x*-*z* plane. In (**c**), the orientation of the quasi-SW C2 field is still in the *x* direction, while that of the C1 field is changed to the *y* direction. The diffracted *σ*^−^ (*σ*^+^) beams are in the *x*-*z* (*y*-*z*) plane. A weak magnetic field is applied in the *z* direction to set the quantization axis of atoms. Note that, for illustration purpose, we exaggerate the misalignment angle between the C1′ and C1″ (C2′ and C2″) beams. In fact, to construct an EIPG in the Raman-Nath regime, the misalignment angles should be very small (typically a few mrad, see details in the Discussion section).

To realize independent control over the *σ*^±^ components of a weak probe field, we here adopt ultracold ^87^Rb atoms as a sample medium and construct a five-level Ξ-Λ system using realistic energy levels of Rb atoms as shown in Fig. 1(a). Initially, the atomic population is optically pumped to |1〉 which serves as the common ground state of the Ξ and Λ configurations. The *σ*^+^ component interacts with the |1〉 ↔ |2〉 transition with a single-photon detuning ${{\rm{\Delta }}}_{{\sigma }^{+}}$, while the *σ*^−^ component interacts with the |1〉 ↔ |3〉 transition with a single-photon detuning ${{\rm{\Delta }}}_{{\sigma }^{-}}$. Two coupling fields (C1 and C2) drive the |2〉 ↔ |4〉 and |3〉 ↔ |5〉 transitions with single-photon detunings Δ_C1_ and Δ_C2_, respectively. Thus, the |1〉 ↔ |2〉 ↔ |4〉 transitions constitute the Λ subsystem and the |1〉 ↔ |3〉 ↔ |5〉 transitions constitute the Ξ subsystem. Note that, due to the dipole selection rule, the atomic transition between |5*P*_1/2_, *F* = 2, *m*_*F*_ = 0〉 and |5*S*_1/2_, *F* = 2, *m*_*F*_ = 0〉 is forbidden and thus cannot interact with the *π*-polarized C1 field^31^.

In principle, we can use standard semiclassical theory to investigate the light-atom interactions in our scheme^32,33^. The effective interaction Hamiltonian of the system within the dipole approximation and the rotating-wave approximation can be written as

1 $$\begin{array}{rcl}{H}_{{\rm{int}}} & = & -\hslash [{{\rm{\Delta }}}_{{\sigma }^{-}}\mathrm{|3}\rangle \langle \mathrm{3|}+({{\rm{\Delta }}}_{{\sigma }^{-}}+{{\rm{\Delta }}}_{{\rm{C2}}})\mathrm{|5}\rangle \langle \mathrm{5|}\\  &  & +{{\rm{\Delta }}}_{{\sigma }^{+}}\mathrm{|2}\rangle \langle \mathrm{2|}+({{\rm{\Delta }}}_{{\sigma }^{+}}-{{\rm{\Delta }}}_{{\rm{C1}}})\mathrm{|4}\rangle \langle \mathrm{4|}]\\  &  & -\frac{\hslash }{2}({{\rm{\Omega }}}_{{\sigma }^{-}}\mathrm{|3}\rangle \langle \mathrm{1|}+{{\rm{\Omega }}}_{{\rm{C2}}}\mathrm{|5}\rangle \langle \mathrm{3|}\\  &  & +{{\rm{\Omega }}}_{{\sigma }^{+}}\mathrm{|2}\rangle \langle \mathrm{1|}+{{\rm{\Omega }}}_{{\rm{C1}}}\mathrm{|2}\rangle \langle \mathrm{4|}+{\rm{H}}.{\rm{c}}.),\end{array}$$

where the frequency detunings are ${{\rm{\Delta }}}_{{\sigma }^{+}}={\omega }_{{\sigma }^{+}}-{\omega }_{21}={\omega }_{{\rm{p}}}-{\omega }_{21}$, ${{\rm{\Delta }}}_{{\sigma }^{-}}={\omega }_{{\sigma }^{-}}-{\omega }_{31}={\omega }_{{\rm{p}}}-{\omega }_{31}$, Δ_C1_ = *ω*_C1_ − *ω*_24_, and Δ_C2_ = *ω*_C2_ − *ω*_53_, the Rabi frequencies are ${{\rm{\Omega }}}_{{\sigma }^{+}}={\mu }_{12}{E}_{{\sigma }^{+}}/\hslash$, ${{\rm{\Omega }}}_{{\sigma }^{-}}={\mu }_{13}{E}_{{\sigma }^{-}}/\hslash$, Ω_C1_ = *μ*_42_*E*_C1_/*ħ*, and Ω_C2_ = *μ*_35_*E*_C2_/*ħ*. Note that *ω*_p_, *ω*_C1_, and *ω*_C2_ are the frequencies of the light fields, *ω*_21_, *ω*_31_, *ω*_24_, and *ω*_53_ are the corresponding atomic transition frequencies, and *μ*_12_, *μ*_13_, *μ*_42_, and *μ*_35_ are the transition dipole moments. To describe the spontaneous emission of the excited states (|2〉, |3〉, and |5〉) and the dephasing process of the ground states (|1〉 and |4〉), we introduce the phenomenological decay rate Γ_*i*_ for each atomic level |*i*〉. Therefore, the equations of motion for the probability amplitudes of the atomic wave function $|\psi (t)\rangle ={\sum }_{i=1}^{5}\,{a}_{i}(t)|i\rangle$ can be given by

2 $${\dot{a}}_{1}=(-{{\rm{\Gamma }}}_{1}\mathrm{/2)}{a}_{1}+i({{\rm{\Omega }}}_{{\sigma }^{+}}{a}_{2}+{{\rm{\Omega }}}_{{\sigma }^{-}}{a}_{3})/2,$$

3 $${\dot{a}}_{2}=(-{{\rm{\Gamma }}}_{2}\mathrm{/2}+i{{\rm{\Delta }}}_{{\sigma }^{+}}){a}_{2}+i({{\rm{\Omega }}}_{{\sigma }^{+}}{a}_{1}+{{\rm{\Omega }}}_{{\rm{C1}}}{a}_{4})/2,$$

4 $${\dot{a}}_{3}=(-{{\rm{\Gamma }}}_{3}\mathrm{/2}+i{{\rm{\Delta }}}_{{\sigma }^{-}}){a}_{3}+i({{\rm{\Omega }}}_{{\sigma }^{-}}{a}_{1}+{{\rm{\Omega }}}_{{\rm{C2}}}{a}_{5})/2,$$

5 $${\dot{a}}_{4}=[-{{\rm{\Gamma }}}_{4}\mathrm{/2}+i({{\rm{\Delta }}}_{{\sigma }^{+}}-{{\rm{\Delta }}}_{{\rm{C1}}})]{a}_{4}+i{{\rm{\Omega }}}_{{\rm{C1}}}{a}_{2}/2,$$

6 $${\dot{a}}_{5}=[-{{\rm{\Gamma }}}_{5}\mathrm{/2}+i({{\rm{\Delta }}}_{{\sigma }^{-}}+{{\rm{\Delta }}}_{{\rm{C2}}})]{a}_{5}+i{{\rm{\Omega }}}_{{\rm{C2}}}{a}_{3}/2,$$

where, for simplicity, all the Rabi frequencies are taken to be real. Because the probe light field is very weak, it is reasonable to believe that the atomic population remains in the initial state |1〉, which means *a*_1_ ≈ 1. Based on the steady-state solutions (${\dot{a}}_{i}=0$, *i* = 1, …, 5) of the above Eqs (2)–(6), we can achieve the linear optical response of the EIT medium under the condition ${{\rm{\Omega }}}_{{\sigma }^{\pm }}$ $\ll$ Ω_C1_, Ω_C2_. The linear susceptibilities read

7 $${\chi }_{{\sigma }^{+}}=\tfrac{{n}_{{\rm{a}}}{\mu }_{12}^{2}}{\hslash {\varepsilon }_{0}{{\rm{\Omega }}}_{{\sigma }^{+}}}{a}_{2}{a}_{1}^{\ast }=\tfrac{{n}_{a}{\mu }_{12}^{2}}{\hslash {\varepsilon }_{0}}\tfrac{i[{{\rm{\Gamma }}}_{4}\mathrm{/2}-i({{\rm{\Delta }}}_{{\sigma }^{+}}-{{\rm{\Delta }}}_{{\rm{C1}}})]}{({{\rm{\Gamma }}}_{2}\mathrm{/2}-i{{\rm{\Delta }}}_{{\sigma }^{+}})\,[{{\rm{\Gamma }}}_{4}\mathrm{/2}-i({{\rm{\Delta }}}_{{\sigma }^{+}}-{{\rm{\Delta }}}_{{\rm{C1}}})]+{{\rm{\Omega }}}_{{\rm{C1}}}^{2}/4},$$

8 $${\chi }_{{\sigma }^{-}}=\tfrac{{n}_{{\rm{a}}}{\mu }_{13}^{2}}{\hslash {\varepsilon }_{0}{{\rm{\Omega }}}_{{\sigma }^{-}}}{a}_{3}{a}_{1}^{\ast }=\tfrac{{n}_{a}{\mu }_{13}^{2}}{\hslash {\varepsilon }_{0}}\tfrac{i[{{\rm{\Gamma }}}_{5}\mathrm{/2}-i({{\rm{\Delta }}}_{{\sigma }^{-}}+{{\rm{\Delta }}}_{{\rm{C2}}})]}{({{\rm{\Gamma }}}_{3}\mathrm{/2}-i{{\rm{\Delta }}}_{{\sigma }^{-}})\,[{{\rm{\Gamma }}}_{5}\mathrm{/2}-i({{\rm{\Delta }}}_{{\sigma }^{-}}+{{\rm{\Delta }}}_{{\rm{C2}}})]+{{\rm{\Omega }}}_{{\rm{C2}}}^{2}/4},$$

where *n*_a_ is the atom number density and the nonlinear susceptibilities are ignored. It is clearly seen in Eqs (7) and (8) that the *σ*^±^ components of the probe field can be decoupled and thus be independently manipulated by dynamically adjusting the two coupling (C1 and C2) fields, which could greatly improve the flexibility for coherent polarization control of photons. This fact also represents a major advantage of our EIPG scheme.

Moreover, because, unlike a perfect SW, the intensity of quasi-SWs in the C1 and C2 coupling fields does not vanish at the quasi-nodal positions [see Fig. 1(b,c)]^34^, the weak-probe-field approximation (i.e., ${{\rm{\Omega }}}_{{\sigma }^{\pm }}$ $\ll$ Ω_C1_, Ω_C2_) can be maintained in the entire interacting region, which also ensures the validity of Eqs (7) and (8) for the linear susceptibilities in the atomic medium. In details, the expressions of the Rabi frequencies of quasi-SW C1 and C2 fields in Eqs (7) and (8) take the forms of

9 $${{\rm{\Omega }}}_{{\rm{C1}}}^{2}(x)=({{\rm{\Omega }}}_{{\rm{C1}}^{\prime} }^{2}+{{\rm{\Omega }}}_{{\rm{C1}}^{\prime\prime} }^{2})+2{{\rm{\Omega }}}_{{\rm{C1}}^{\prime} }{{\rm{\Omega }}}_{{\rm{C1}}^{\prime\prime} }\,\cos \,(2\pi x/G1),$$

10 $${{\rm{\Omega }}}_{{\rm{C2}}}^{2}(x)=({{\rm{\Omega }}}_{{\rm{C2}}^{\prime} }^{2}+{{\rm{\Omega }}}_{{\rm{C2}}^{\prime\prime} }^{2})+2{{\rm{\Omega }}}_{{\rm{C2}}^{\prime} }{{\rm{\Omega }}}_{{\rm{C2}}^{\prime\prime} }\,\cos \,(2\pi x/G2),$$

for the one-dimensional (1D) EIPG shown in Fig. 1(b), and

11 $${{\rm{\Omega }}}_{{\rm{C1}}}^{2}(y)=({{\rm{\Omega }}}_{{\rm{C1}}^{\prime} }^{2}+{{\rm{\Omega }}}_{{\rm{C1}}^{\prime\prime} }^{2})+2{{\rm{\Omega }}}_{{\rm{C1}}^{\prime} }{{\rm{\Omega }}}_{{\rm{C1}}^{\prime\prime} }\,\cos \,(2\pi y/G1),$$

12 $${{\rm{\Omega }}}_{{\rm{C2}}}^{2}(x)=({{\rm{\Omega }}}_{{\rm{C2}}^{\prime} }^{2}+{{\rm{\Omega }}}_{{\rm{C2}}^{\prime\prime} }^{2})+2{{\rm{\Omega }}}_{{\rm{C2}}^{\prime} }{{\rm{\Omega }}}_{{\rm{C2}}^{\prime\prime} }\,\cos \,(2\pi x/G2),$$

for the two-dimensional (2D) case shown in Fig. 1(c), respectively. The spatial periods of the quasi-SW C1 and C2 fields are *G*1 and *G*2. The terms Ω_C1′_, Ω_C1″_, Ω_C2′_, and Ω_C2″_ represent the Rabi frequencies of the four laser beams that generate the quasi-SW C1 and C2 fields, where we have Ω_C1′_ ≠ Ω_C1″_ and Ω_C2′_ ≠ Ω_C2″_.

### Fraunhofer diffraction of 1D EIPG

For the probe field travelling through a thin (Raman-Nath) 1D EIPG in our scheme, its propagation is governed by Maxwell’s equation which can be simplified as $\partial {E}_{{\sigma }^{\pm }}/\partial z=i{k}_{{\rm{p}}}{\chi }_{{\sigma }^{\pm }}{E}_{{\sigma }^{\pm }}/2$ under the paraxial approximation, where the transverse terms ${\partial }^{2}{E}_{{\sigma }^{\pm }}/\partial {x}^{2}$ and ${\partial }^{2}{E}_{{\sigma }^{\pm }}/\partial {y}^{2}$ are ignored^4,12^. Assuming that the probe field is a plane wave incident in the *z* direction (i.e., ${E}_{{\sigma }^{\pm }}^{{\rm{in}}}=1$), for the 1D case shown in Fig. 1(b), the far-field (Fraunhofer) diffraction amplitudes can be given by 1D Fourier transform^35^, i.e.,

13 $${E}_{{\sigma }^{\pm }}({\theta }_{{\sigma }^{\pm }})\propto {\int }_{-\infty }^{+\infty }\,{E^{\prime} }_{{\sigma }^{\pm }}(x)\,\exp \,(-i{k}_{{\rm{p}}}x\,\sin \,{\theta }_{{\sigma }^{\pm }})\,{\rm{d}}x={\int }_{-\infty }^{+\infty }\,{t}_{{\sigma }^{\pm }}(x)\,\exp \,(-i{k}_{{\rm{p}}}x\,\sin \,{\theta }_{{\sigma }^{\pm }})\,{\rm{d}}x,$$

where $${E^{\prime} }_{{\sigma }^{\pm }}(x)={E}_{{\sigma }^{\pm }}^{{\rm{in}}}{t}_{{\sigma }^{\pm }}(x)$$ are the transmitted *σ*^±^ components immediately behind the grating, $${t}_{{\sigma }^{\pm }}(x)=$$$$\exp (i{k}_{{\rm{p}}}{\chi }_{{\sigma }^{\pm }}d/2)$$ are the amplitude transmission functions, the susceptibilities $${\chi }_{{\sigma }^{\pm }}$$ are periodic functions of *x*, and $${\theta }_{{\sigma }^{\pm }}$$ are the diffraction angles of *σ*^±^ components with respect to the *z* direction. Note that we here ignore the grating height (*h* = 1 mm) in the *y* direction because there is no grating period in such direction in the 1D case, which has very little influence on the diffraction in the *x* direction.

If there are ${M}_{{\sigma }^{+}}$ (an integer) spatial periods in the grating for the *σ*^+^ component (i.e., the grating width $w={M}_{{\sigma }^{+}}{G}_{1}$), the diffraction intensity based on Eq. (13) becomes

14 $${I}_{{\sigma }^{+}}({\theta }_{{\sigma }^{+}})={|\frac{\sin ({M}_{{\sigma }^{+}}{G}_{1}{k}_{{\rm{p}}}\sin {\theta }_{{\sigma }^{+}}/2)}{{M}_{{\sigma }^{+}}\sin ({G}_{1}{k}_{{\rm{p}}}\sin {\theta }_{{\sigma }^{+}}/2)}|}^{2}\times {|{E}_{{\sigma }^{+}}^{{\rm{s}}}({\theta }_{{\sigma }^{+}})|}^{2}.$$

The quantity ${E}_{{\sigma }^{+}}^{{\rm{s}}}({\theta }_{{\sigma }^{+}})$ stands for the far-field diffraction amplitude stemming from one single grating period, which reads

15 $${E}_{{\sigma }^{+}}^{{\rm{s}}}({\theta }_{{\sigma }^{+}})={\tilde{c}}_{{\sigma }^{+}}\,{\int }_{0}^{{G}_{1}}\,{t}_{{\sigma }^{+}}(x)\,\exp \,(-i{k}_{{\rm{p}}}x\,\sin \,{\theta }_{{\sigma }^{+}})\,{\rm{d}}x,$$

where ${\tilde{c}}_{{\sigma }^{+}}$ is the normalization coefficient in the absence of modulation.

To characterize the diffraction ability of EIPG for the *σ*^+^ component, we calculate the diffraction intensity along the 1st-order diffraction angle ${\theta }_{{\sigma }^{+}}^{{\rm{1st}}}=\arcsin ({\lambda }_{{\rm{p}}}/G1)$, which is

16 $${I}_{{\sigma }^{+}}^{{\rm{1st}}}={|{E}_{{\sigma }^{+}}^{{\rm{s}}}({\theta }_{{\sigma }^{+}}^{{\rm{1st}}})|}^{2}.$$

More importantly, to thoroughly evaluate the EIPG performance for polarization manipulation, we should also derive a series of related expressions for the *σ*^−^ component by replacing the subscript *σ*^+^ with *σ*^−^ and the grating period *G*1 with *G*2 in Eqs (14–16). In this way, one can directly compare the diffraction properties of EIPG for different polarization components.

## Results

### Parameter settings

Before embarking on detailed diffraction calculations, we should prepare some necessary parameters in our scheme. In experiment, cold atomic cloud with high optical depth can be prepared in a magneto-optical trap (MOT)^36^. Thus, we assume the atomic number density *n*_a_ ≈ 10^12^/cm^3^. Considering the finite sizes of laser beams and atomic cloud, the spatial region for effective light-atom interactions is assumed to be roughly within a cubic range of 1 × 1 × 1 mm^3^ in the atomic cloud, which also characterizes the width *w* (=1 mm), height *h* (=1 mm), and thickness *d* (=1 mm) of the induced grating.

Because a “thin” grating instead of a “thick” (Bragg) grating is considered in our scheme, operating in the Raman-Nath limit, we adopt a criteria in optical holography to determine whether the grating is thin or thick^16,17^. For the criteria, a dimensionless factor *Q* = 2*πλd*/(*n*_*i*_*G*) < 1 is required for a thin Raman-Nath grating, where *λ* is the wavelength of the incident light field, *d* is the thickness of the grating, *n*_*i*_ is the refractive index of the medium, and *G* is the grating period. In our EIT system, we have *λ* = *λ*_p_ ≈ 795 nm, *d* = 1 mm, and *n*_*i*_ ≈ 1, which lead to a restriction for the grating periods (i.e., {*G*_1_, *G*_2_} > 70.7 *μ*m). Therefore, to satisfy this restriction in the following numerical calculations, we assume that the grating periods induced by the C1 and C2 quasi-SW fields are *G*1 = 125 *μ*m and *G*2 = 100 *μ*m, respectively.

Moreover, as an example, we here choose the Zeeman sublevel |10*S*_1/2_, *F* = 2, *m*_*F*_ = +1〉 as the highest excited state |5〉. For the 10*s* level of ^87^Rb, we can have the decay rate Γ_5_ = 2*π* × 0.37 MHz corresponding to the lifetime of about 430 ns, the hyperfine splitting ${{\rm{\Delta }}^{\prime\prime} }_{{\rm{HFS}}}=112.5$ MHz, and the wavelength of C2 field (the |3〉 ↔ |5〉 transition) *λ*_C2_ = 532.24 nm^37,38^. For the parameters related to other energy levels, we have the wavelength of C1 field *λ*_C1_ = 795 nm (the |2〉 ↔ |4〉 transition), the decay rates Γ_1_ = Γ_4_ = 2*π* × 3 kHz, and Γ_2_ = Γ_3_ = Γ = 2*π* × 6 MHz^31^. Also, the single-photon detunings of the *σ*^±^ components satisfy the relationship ${{\rm{\Delta }}}_{{\sigma }^{-}}={{\rm{\Delta }}}_{{\sigma }^{+}}+2{\mu }_{{\rm{B}}}{g}_{F}B$, where *μ*_B_ is the Bohr magneton, *g*_*F*_ is the hyperfine Landé *g*-factor, *μ*_B_*g*_*F*_ = 0.23 MHz/G for the 5*P*_1/2_ state of ^87^Rb, and *B* is the weak magnetic field in gauss^31^.

Additionally, for simplicity, we here assume that the coupling fields are always resonant with the corresponding atomic transitions, i.e., Δ_C1_ = Δ_C2_ = 0. If we use a weak magnetic field (e.g., *B* = 10 mG) to set the quantization axis of atoms, the two states |2〉 and |3〉 can be regarded to be nearly degenerate when the energy splitting (2 *μ*_B_*g*_*F*_*B* = 4.6 KHz) is much smaller than the EIT linewidth estimated by ${{\rm{\Omega }}}_{{\rm{C1}}}^{2}/{{\rm{\Gamma }}}_{2}$ and ${{\rm{\Omega }}}_{{\rm{C2}}}^{2}/{{\rm{\Gamma }}}_{3}$. Under such condition, the frequency detunings of the *σ*^±^ components are set to be approximately equal (i.e., ${{\rm{\Delta }}}_{{\sigma }^{+}}$ ≈ ${{\rm{\Delta }}}_{{\sigma }^{-}}$) in the numerical calculations. To characterize the periodic phase and amplitude modulations, we also define the phase modulation as ${\rm{\Phi }}={k}_{{\rm{p}}}{\rm{Re}}({\chi }_{{\sigma }^{\pm }})d/2$ and the intensity transmission rate as $T=\exp [-{k}_{{\rm{p}}}\,{\rm{Im}}({\chi }_{{\sigma }^{\pm }})d]$.

### Numerical results for 1D EIPG

**Figure 2:**
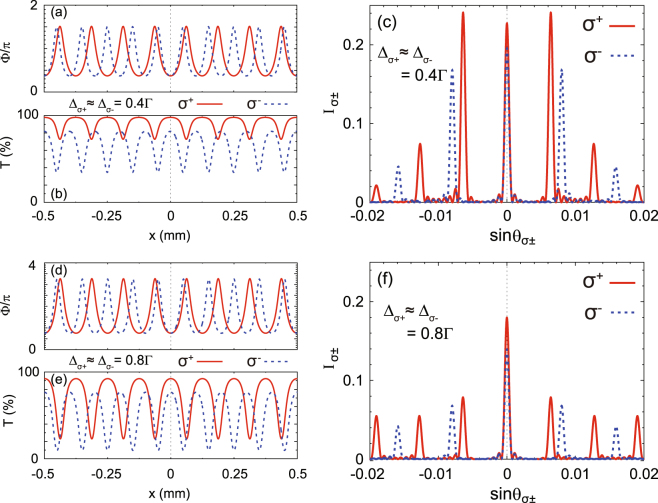
Spatial variations of the phase modulation Φ (**a**) and the intensity transmission *T* (**b**) for ${{\rm{\Delta }}}_{{\sigma }^{+}}\approx {{\rm{\Delta }}}_{{\sigma }^{-}}=0.4{\rm{\Gamma }}$, which can produce an EIPG to diffract the probe *σ*^±^ components through the atomic ensemble. The grating width in the *x* direction is *w* = 1 mm. (**c**) The normalized diffraction intensities ${I}_{{\sigma }^{\pm }}$ with respect to the diffraction angles $\sin \,{\theta }_{{\sigma }^{\pm }}$ for ${{\rm{\Delta }}}_{{\sigma }^{+}}\approx {{\rm{\Delta }}}_{{\sigma }^{-}}=0.4{\rm{\Gamma }}$. For ${{\rm{\Delta }}}_{{\sigma }^{+}}\approx {{\rm{\Delta }}}_{{\sigma }^{-}}=0.8{\rm{\Gamma }}$, we have the spatial variations of Φ in (**d**), *T* in (**e**), and ${I}_{{\sigma }^{\pm }}$ in (**f**). Note that we fix the intensities of the coupling fields as Ω_C1′_ = Ω_C2′_ = 7.5Γ and Ω_C1″_ = Ω_C2″_ = 2.5Γ in Fig. 2.

**Figure 3:**
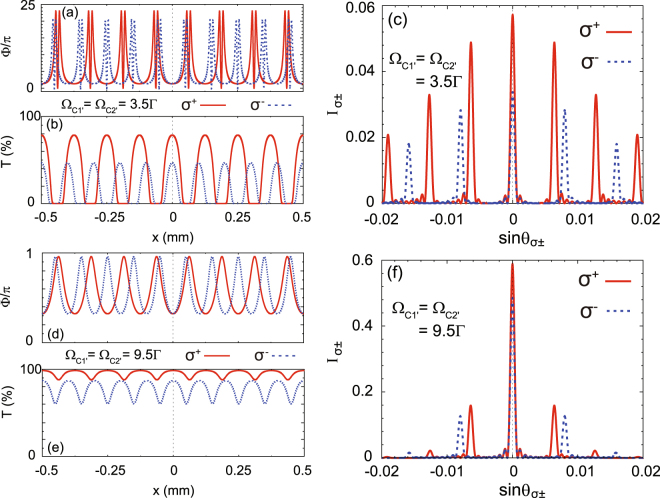
Spatial variations of the phase modulation Φ (**a**) and the intensity transmission *T* (**b**) for relatively weak quasi-SW coupling fields (Ω_C1′_ = Ω_C2′_ = 3.5Γ) in the atomic ensemble. The grating width in the *x* direction is *w* = 1 mm. The normalized diffraction intensities ${I}_{{\sigma }^{\pm }}$ are shown in (**c**). For strong quasi-SW coupling fields (Ω_C1′_ = Ω_C2′_ = 9.5Γ), the spatial variations of Φ, *T*, and ${I}_{{\sigma }^{\pm }}$ are depicted in (**d**–**f**), respectively. Note that we set Ω_C1″_ = Ω_C2″_ = 2.5Γ and ${{\rm{\Delta }}}_{{\sigma }^{+}}\approx {{\rm{\Delta }}}_{{\sigma }^{-}}=0.5{\rm{\Gamma }}$ in Fig. 3.

By varying the frequency detunings of the probe field or the intensities of the quasi-SW coupling (C1 and C2) fields and setting different grating periods in the C1 and C2 fields, the susceptibilities of the EIT system can be strongly modified. Therefore, it is possible to independently control and spatially separate the *σ*^±^ components of the probe field. In Figs 2 and 3, it is clearly seen that the high-order diffraction modes of the *σ*^±^ components can be separated in space because they experience different grating periods [i.e., *G*1 = 125 *μ*m for *σ*^+^ and *G*2 = 100 *μ*m for *σ*^−^ in Eqs (9) and (10)]. To be specific, in Fig. 2, we fix the intensities of the coupling fields as Ω_C1′_ = Ω_C2′_ = 7.5Γ and Ω_C1″_ = Ω_C2″_ = 2.5Γ in Eqs (9) and (10). When the probe frequency detunings are small (i.e., ${{\rm{\Delta }}}_{{\sigma }^{+}}\approx {{\rm{\Delta }}}_{{\sigma }^{-}}=0.4{\rm{\Gamma }}$), the phase modulation strength ΔΦ = (Φ_max_ − Φ_min_) is slightly greater than *π* (1.13*π* for *σ*^−^ and 1.14*π* for *σ*^+^) and the intensity transmission is higher than 34% for *σ*^−^ and 72% for *σ*^+^ [Fig. 2(a,b)]. Therefore, the EIT system mainly works as a phase grating, which means that more incident energy is scattered into the higher-order diffraction modes [Fig. 2(c)]. When the probe frequency detunings are increased to ${{\rm{\Delta }}}_{{\sigma }^{+}}\approx {{\rm{\Delta }}}_{{\sigma }^{-}}=0.8{\rm{\Gamma }}$, the phase modulation strength ΔΦ is increased to ~2.5*π* (2.49*π* for *σ*^−^ and 2.51*π* for *σ*^+^) but the intensity transmission rate is greatly lowered where the minimum of *T* is 9.7% for *σ*^−^ and 23% for *σ*^+^ [Fig. 2(d,e)]. Therefore, the total diffraction efficiency is decreased due to high absorption. But, the intensity distributions of the higher-order diffraction modes are more uniform due to the strong phase modulation [Fig. 2(f)].

Figure 3 illustrates that the intensities of the quasi-SW coupling fields can significantly modify the diffraction patterns when we fix the probe detunings as ${{\rm{\Delta }}}_{{\sigma }^{+}}\approx {{\rm{\Delta }}}_{{\sigma }^{-}}=0.5{\rm{\Gamma }}$. To change the intensities of the quasi-SW coupling fields, we fix the intensities of the C1″ and C2″ laser beams in the C1 and C2 fields as Ω_C1″_ = Ω_C2″_ = 2.5Γ, but tune the intensities of the C1′ and C2′ laser beams. When Ω_C1′_ = Ω_C2′_ = 3.5Γ, the transparency window of the EIT system is relatively narrow, which leads to strong phase modulation (ΔΦ > 20*π* for *σ*^±^) as well as severe absorption [Fig. 3(a,b)]. Therefore, the total diffraction (transmission) efficiency is low (5.7% for the *σ*^+^ central principal maximum and 3.4% for the *σ*^−^ central principal maximum), where more intensity is diffracted into the higher-order modes [Fig. 3(c)]. As a comparison, when Ω_C1′_ = Ω_C2′_ = 9.5Γ, the transparency window of the EIT system is much wider for high quasi-SW intensity. Accordingly, the phase modulation becomes weak (ΔΦ ≈ 0.64*π* for *σ*^±^) but the transmission is high [Fig. 3(d,e)]. The central principal maximum is greatly enhanced to 58.9% for *σ*^+^ and 48.5% for *σ*^−^, and the ±1st-order maximum is 16% for *σ*^+^ and 13% for *σ*^−^ [Fig. 3(f)].

**Figure 4:**
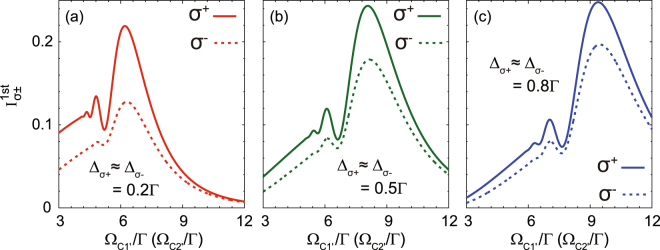
Evolution of the 1st-order diffraction intensity as a function of Ω_C1′_ (Ω_C2′_) with increasing probe detuning, where ${{\rm{\Delta }}}_{{\sigma }^{+}}\approx {{\rm{\Delta }}}_{{\sigma }^{-}}=0.2{\rm{\Gamma }}$ (**a**), 0.5Γ (**b**), and 0.8Γ (**c**). Note that we set Ω_C1″_ = Ω_C2″_ = 2.5Γ.

**Figure 5:**
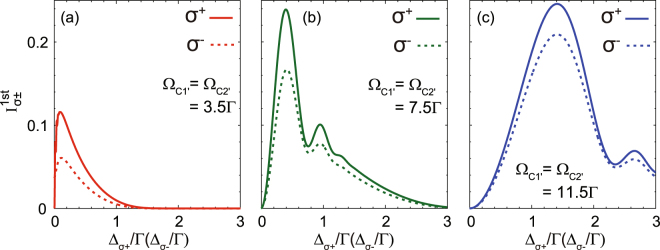
Evolution of the 1st-order diffraction intensity as a function of ${{\rm{\Delta }}}_{{\sigma }^{+}}$ $(\approx {{\rm{\Delta }}}_{{\sigma }^{-}})$ with increasing coupling intensity, where Ω_C1′_ = Ω_C2′_ = 3.5Γ (**a**), 7.5Γ (**b**), and 11.5Γ (**c**). Note that we set Ω_C1″_ = Ω_C2″_ = 2.5Γ.

Because, in general, the intensity of the 1st-order diffraction mode is important to evaluate the optical performance of a grating, we also demonstrate the intensity evolution with respect to the optical parameters of EIPG using Eqs (15) and (16). In Fig. 4, we investigate the 1st-order diffraction intensity as a function of Ω_C1′_ (Ω_C2′_) with increasing probe detunings, where we fix Ω_C1″_ = Ω_C2″_ = 2.5Γ. It is seen that the 1st-order diffraction intensity can be enhanced to 24.8% for *σ*^+^ and 19.7% for *σ*^−^ with higher coupling intensity and larger probe detuning [see Fig. 4(c)]. Moreover, the 1st-order diffraction intensity of the *σ*^+^ component is always higher than that of the *σ*^−^ component for the same EIT parameters. Such a feature originates from the fact that, in our scheme, the *σ*^+^ component is manipulated in a Λ-type EIT subsystem, whereas the *σ*^−^ component is in a Ξ-type EIT subsystem. Due to the higher decoherence rate in the Ξ-type subsystem, its EIT efficiency is usually lower than that of the Λ-type subsystem, thereby leading to lower diffraction efficiency. A similar tendency is also presented in Fig. 5. Higher coupling intensity and larger probe detuning can raise the 1st-order diffraction efficiency to 24.6% for *σ*^+^ and 21% for *σ*^−^ [see Fig. 5(a–c)]. Because under such conditions, the EIPGs act more like a phase grating than an amplitude grating, where the absorption is suppressed.

Additionally, there is one point that should be emphasized concerning our numerical results in Figs 2 and 4, where we use the weak-magnetic-field approximation and nearly degenerate Zeeman sublevels (i.e., ${{\rm{\Delta }}}_{{\sigma }^{+}}$ ≈ ${{\rm{\Delta }}}_{{\sigma }^{-}}$) in the calculations. However, the data in Figs 2 and 4 can also give us a hint to study the *σ*^±^ diffraction intensities with moderate magnetic field by comparing the corresponding curves between different subfigures. For example, we can make a comparison between the diffraction intensity of *σ*^+^ with ${{\rm{\Delta }}}_{{\sigma }^{+}}=0.4{\rm{\Gamma }}$ [red solid curve in Fig. 2(c)] and that of *σ*^−^ with ${{\rm{\Delta }}}_{{\sigma }^{-}}=0.8{\rm{\Gamma }}$ [blue dotted curve in Fig. 2(f)], where the corresponding magnetic field is give by $B=({{\rm{\Delta }}}_{{\sigma }^{-}}-{{\rm{\Delta }}}_{{\sigma }^{+}})/2{\mu }_{{\rm{B}}}{g}_{F}=10.4$ G. In Fig. 4, we can also compare the 1st-order *σ*^±^ diffraction intensities for different frequency detunings, for example, the red solid curve in Fig. 4(a) for *σ*^+^ with ${{\rm{\Delta }}}_{{\sigma }^{+}}=0.2{\rm{\Gamma }}$ and the blue dotted curve in Fig. 4(c) for *σ*^−^ with ${{\rm{\Delta }}}_{{\sigma }^{-}}=0.8{\rm{\Gamma }}$. In this case, the corresponding magnetic field is *B* = 15.6 G. Therefore, our results not only directly compare the *σ*^±^ diffraction under weak magnetic field, but also offer the opportunity to assess the influence of moderate magnetic field on the diffraction of the *σ*^±^ components. This fact also means that magnetic field can greatly increase the tunability of polarization-selective diffraction in our EIPG scheme.

### Numerical results for 2D EIPG

**Figure 6:**
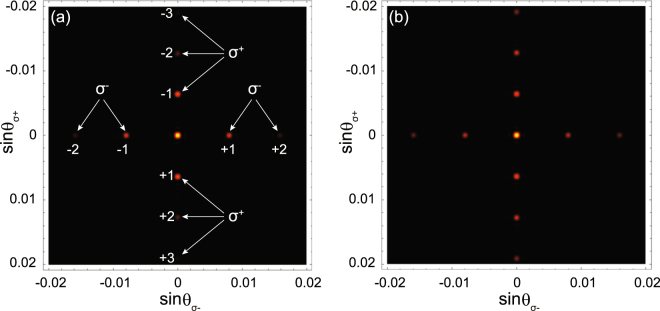
Polarization-resolved far-field (Fraunhofer) diffraction patterns in 2D originating from the experimental setup in Fig. 1(c). The grating width in the *x* direction is *w* = 1 mm and the grating height in the *y* direction is *h* = 1 mm. The *σ*^±^ components can be diffracted either in horizontal or vertical direction, respectively, and are well-separated in space. In (**a**), we use the same EIT parameters as those in Fig. 2(a–c), while (**b**) has the same EIT parameters as those in Fig. 3(a–c). Because we normalize the central intensity maxima in (**a**,**b**), the pictures show the relative intensity distributions.

Because the Ξ-type and Λ-type subsystems are totally independent of each other, we can change the grating orientation in one subsystem to diffract different polarization components in 2D. For example, we can generate two perpendicular grating structures in Fig. 1(c), where the quasi-SW in the C1 field is along the *y* direction described by Eq. (11). Thus, the *σ*^+^ component is diffracted in the *y* direction, whereas the *σ*^−^ component is still diffracted in the *x* direction. Consequently, the *σ*^±^ components can be separated far apart in space, which may show more practicability and flexibility than the 1D case. To do the numerical calculations, we employ the EIT parameters in Figs 2(a–c) and 3(a–c) and Eqs (11) and (12) to perform 2D Fourier transform^35^, where the grating width is *w* = 1 mm in the *x* direction and the grating height is *h* = 1 mm in the *y* direction [see Fig. 1(c)]. The far-field diffraction patterns are indicated in Fig. 6. Here, for brevity, the tedious analytical expressions for the 2D Fourier integral are omitted.

It is well-known that 2D optical diffraction is a fundamental operation for multimode spatial information processing^35,39^. Our 2D EIPG scheme could be combined with other models in atomic EIT systems to find potential applications in polarization-dependent beam splitting and fanning^12–14^, image processing^40–42^, and vortex manipulation^43–45^, which may further improve the capacity and speed for parallel signal processing by all-optical means.

## Discussion

Although a tripod system is usually employed for polarization-dependent photon manipulation^20–22^, the polarization-dependent multibeam diffraction with our EIPG scheme in the Raman-Nath limit cannot be simply realized using a tripod EIT system. The basic challenge is that, in a tripod EIT system, one SW coupling field can only generate one single grating period for both circular polarization *σ*^±^ components of a linearly polarized probe field although the refractive index modulation strengths may be different. According to the scalar diffraction theory^35,39^, the far-field diffraction angle of a thin (Raman-Nath) grating (regardless of an amplitude or phase grating) only depends on the ratio between incident wavelength and grating period. Therefore, for the same diffraction order, the *σ*^±^ components will have the same diffraction angle for the EIG generated in a tripod system. As a result, the transmitted *σ*^±^ components cannot be transversely separated in free space. Namely, for each high diffraction order, one can hardly achieve a pure *σ*^+^ or *σ*^−^ component. However, by constructing a five-level Ξ-Λ system in this work, the *σ*^±^ components can be fully separated in high diffraction orders and the separation distance can be flexibly adjusted by independently changing the grating periods *G*_1_ and *G*_2_ of the two quasi-SW coupling fields.

Recently, 2D periodic structures have been investigated to broaden the applications of EIG systems^11,19^. The function of our 2D EIPG is totally different from these already existing proposals. In ordinary 2D EIGs, the incident probe field directly experiences a 2D grating structure without polarization sensitivity and thus the far-field diffraction pattern is not polarization-resolved. As a comparison, in our 2D EIPG, the *σ*^±^ components of a linearly polarized probe field actually experience different 1D grating structures having perpendicular orientations, respectively. The far-field diffraction pattern is a spatial combination of two independently tunable patterns with different polarizations, which further enriches the diffraction phenomena of EIG systems. More importantly, the 2D EIPG case also clearly shows the advantage of our five-level Ξ-Λ system over the conventional tripod system because it is hard for the tripod system with one single coupling field to independently fan out different polarization components in 2D in the Raman-Nath limit.

To produce the quasi-SWs in the C1 and C2 coupling fields, we propose the possible experimental setups in Fig. 1(b,c). We first assume that the probe field propagates in the *z* direction, and a weak magnetic field is also applied in the same direction to set the quantization axis of atoms. Two *π*-polarized laser beams (C1′ and C1″) with unequal intensity in the *x*-*y* plane can interfere to form the quasi-SW C1 field whose polarization direction is parallel to the *z* direction. By adjusting the misalignment angle *ϕ*_1_ between the two lasers, we can change the grating period *G*_1_ = *λ*_C1_/[2 sin (*ϕ*_1_/2)] in the C1 field where *λ*_C1_ is the wavelength of C1 field. Similarly, two *σ*^+^ -polarized laser beams (C2′ and C2″) with unequal intensity in the *x*-*z* plane can form the quasi-SW C2 field. Thus, the grating period of C2 field is given by *G*_2_ = *λ*_C2_/[2 sin (*ϕ*_2_/2)], where *ϕ*_2_ is the misalignment angle and *λ*_C2_ is the wavelength of C2 field. Without loss of generality, we assume that the coordinate axes *y* and *z* in Fig. 1(b) [or, *x* and *z* in Fig. 1(c)] are the bisectors of the misalignment angles *ϕ*_1_ and *ϕ*_2_, respectively. Thus, the orientation of the quasi-SW C1 field is along the *x* direction in Fig. 1(b) and along the *y* direction in Fig. 1(c), while that of the C2 field is always along the *x* direction. Consequently, two sets of polarization-resolved grating structures could be independently created in 1D and 2D.

In Fig. 1(b,c), the misalignment angle *ϕ*_1_ (*ϕ*_2_) between the C1′ and C1″ (C2′ and C2″) beams is actually very small to construct the EIPG in the Raman-Nath regime. For the grating period *G*1 = 125 *μ*m in the C1 field (*λ*_C1_ = 795 nm), we have *ϕ*_1_ = 6.36 mrad. However, the situation for the C2 field is slightly complicated. For *G*2 = 100 *μ*m in the C2 field (*λ*_C2_ = 532.24 nm), we have *ϕ*_2_ = 5.32 mrad. This also means that the *σ*^+^-polarized C2′ and C2″ beams are not strictly parallel to the magnetic field, which can give rise to other polarization components (e.g., *σ*^−^ and *π*) in the resulting quasi-SW C2 field. One can analytically derive the expression of the quasi-SW C2 field and find that the peak intensity ratio between the *σ*^+^-, *σ*^−^-, and *π*-components is given by {[1 + cos (*ϕ*_2_/2)]/2}^2^ : {[1 – cos (*ϕ*_2_/2)]/2}^2^ : [sin (*ϕ*_2_/2)]^2^/2 = 0.9999964622 : 3.129 × 10^−12^ : 3.538 × 10^−6^, where *ϕ*_2_/2 = 2.66 mrad is the angle between each C2 beam (i.e., C2′ or C2″) and the quantization (*z*) axis (the bisector of *ϕ*_2_). Therefore, in our scheme, the *σ*^+^-polarized component plays a highly predominant role in the quasi-SW C2 field and other polarization components can be ignored.

In practice, to suppress the influence of MOT on the EIT system in a cold atomic ensemble, the probe light field should be turned on after the trap is switched off. Therefore, the EIT measurement can be performed using a time sequence. Such a technique has been widely exploited in the EIG experiments with cold atoms^5,7^, where the 1/*e* lifetime of the MOT after switching off the trapping beams is of the order of 2 ms^7^. Thus, the duration of the probe light field could be a few hundreds of *μ*s, which leads to the linewidth of the probe field less than 10 kHz. Such a linewidth is much smaller than the EIT linewidth in our work. For example, the narrowest EIT linewidth is given by the data in Fig. 4, where we have Ω_C1′_ = Ω_C2′_ = 3Γ and Ω_C1″_ = Ω_C2″_ = 2.5Γ at the leftmost side of each subfigure. Based on Eqs (9) and (10), we have Ω_C1_ = Ω_C2_ = 0.5Γ at the quasi-nodal position and the EIT linewidth is thus estimated by ${{\rm{\Omega }}}_{{\rm{C1}}}^{2}/{\rm{\Gamma }}={{\rm{\Omega }}}_{{\rm{C2}}}^{2}/{\rm{\Gamma }}=0.25{\rm{\Gamma }}=1.5$ MHz. Therefore, the probe field can be treated as nearly monochromatic and our theoretical model is still valid in the cold atoms.

To obtain the linear susceptibilities [i.e., Eqs (7) and (8)] and thus enable the decoupling between the *σ*^±^ components of the probe field, the total photon number in the probe field should be much smaller than the atom number in the interacting region to maintain the atomic population in the initial state |1〉. The total photon number can be given by ${{\mathscr{N}}}_{{\rm{ph}}}={I}_{{\rm{p}}}A\tau /(\hslash {\omega }_{{\rm{p}}})$, where ${I}_{{\rm{p}}}={\varepsilon }_{0}c{E}_{{\rm{p}}}^{2}/2$ is the intensity of the probe field, *ε*_0_ is the vacuum permittivity, *c* is the vacuum light speed, *A* is the area of the cross section of the interacting region, and *τ* is the duration time of the probe field. Using the parameters in the “Parameter settings” subsection, we have *A* = 1 × 1 mm^2^ and the atom number ${{\mathscr{N}}}_{{\rm{a}}}={n}_{{\rm{a}}}\times V={10}^{9}$ in the interacting region where *n*_a_ = 10^12^/cm^3^ and *V* = 1 mm^3^. Also, we assume *τ* ~ 100 *μ*s based on the linewidth analysis in the above paragraph. Due to the limit of ${{\mathscr{N}}}_{{\rm{ph}}}\ll {{\mathscr{N}}}_{{\rm{a}}}$, the range of the total Rabi frequency of the probe field can be given by ${{\rm{\Omega }}}_{{\rm{p}}}={\mu }_{{\rm{D1}}}{E}_{{\rm{p}}}/\hslash \ll 0.277{\rm{\Gamma }}$, where *μ*_D1_(=*μ*_12_ = *μ*_13_) = 2.54 × 10^−29^ C · m is the dipole moment of the ^87^Rb D1 transition (5*S*_1/2_ ↔ 5*P*_1/2_)^31^. Such a result is also consistent with the condition ${{\rm{\Omega }}}_{{\sigma }^{\pm }}$ $\ll$ Ω_C1_, Ω_C2_ which is another important prerequisite to derive Eqs (7) and (8). Hence, in our Ξ-Λ EIT system, the probe *σ*^±^ components can be decoupled under appropriate conditions.

The low absorption and high diffraction efficiency of our EIPG scheme may also find potentials in quantum information processing. For example, when a single probe photon with linear polarization is incident, it is possible to obtain the polarization and space entangled multiple Fock states in the far field, such as $|{{\rm{\Psi }}}_{{\rm{far}}}\rangle ={\sum }_{i=-n}^{+n}\,{b}_{i}|{0}_{{\sigma }^{+},{\theta }_{-n}}\rangle \cdots |{1}_{{\sigma }^{+},{\theta }_{i}}\rangle \cdots |{0}_{{\sigma }^{+},{\theta }_{+n}}\rangle |{0}_{{\sigma }^{-},{\theta }_{-m}}\rangle \cdots |{0}_{{\sigma }^{-},{\theta }_{+m}}\rangle +{\sum }_{j=-m}^{m}\,{b}_{j}|{0}_{{\sigma }^{+},{\theta }_{-n}}\rangle \cdots |{0}_{{\sigma }^{+},{\theta }_{+n}}\rangle |{0}_{{\sigma }^{-},{\theta }_{-m}}\rangle \cdots$  $|{1}_{{\sigma }^{-},{\theta }_{j}}\rangle \cdots |{0}_{{\sigma }^{-},{\theta }_{+m}}\rangle$ where the subscripts (*σ*^+^, *θ*_*i*_) and (*σ*^−^, *θ*_*j*_) denote the *i*th-order diffraction angle of the *σ*^+^ component and the *j*th-order diffraction angle of the *σ*^−^ component, respectively. Therefore, our EIPG scheme using the polarization degree of freedom can increase the capacity of multichannel optical devices for quantum information processing.

Note that we choose the 10*s* level only as an example to design the EIPG. Actually, other lowly excited *s* levels (e.g., 6*S*_1/2_) can also be used as the state |5〉 in our scheme. For highly excited *s* levels in Rydberg atoms, the dipole-dipole interactions can generate strong optical nonlinearity^46,47^, which is beyond our theoretical model [see Eq. (1)] and will be considered in future work.

In summary, we have studied the possibility of generating a polarization-resolved Raman-Nath grating in an EIT medium with ultracold ^87^Rb atoms. Considering the multiple Zeeman sublevels in ^87^Rb, we design a five-level Ξ-Λ system and the polarization-dependent optical susceptibilities of the system are theoretically derived. By adjusting the EIT parameters, including the probe frequency detunings and the quasi-SW coupling light fields, we numerically calculate the far-field (Fraunhofer) diffraction distributions of a probe light field normally incident on the EIT medium. Our results show that the probe *σ*^±^ components can be decoupled and independently diffracted in 1D and 2D. Such fact means that the two polarization components can be flexibly and efficiently controlled based on EIPG systems, which may increase the channels and enhance the performance of all-optical devices and networks. This idea could also be extended to complicated optical structures, such as images and vortices, offering a versatile platform for polarization-selective spatial multimode information processing in EIT media.
